# Elicitation of quantitative, choice-based preferences for Person-Centered Care among People living with Dementia in comparison to physicians’ judgements in Germany: study protocol for the mixed-methods *PreDemCare*-study

**DOI:** 10.1186/s12877-022-03238-6

**Published:** 2022-07-08

**Authors:** Wiebke Mohr, Anika Rädke, Bernhard Michalowsky, Wolfgang Hoffmann

**Affiliations:** 1 German Center for Neurodegenerative Diseases e.V. (DZNE), Site Rostock / Greifswald, Ellernholzstraße 1-2, 17487 Greifswald, Germany; 2 Institute for Community Medicine, University Medicine Greifswald (UMG), Greifswald, Germany

**Keywords:** Patient preferences, Dementia, Person-centered care, Patient-centered care, Patient participation, Analytic hierarchy process, Multi-criteria decision analysis, Protocol, Decision-making

## Abstract

**Background:**

Person-Centered-Care (PCC) requires knowledge about patient preferences. Among People-living-with-Dementia (PlwD) data on quantitative, choice-based preferences, which would allow to *quantify, weigh* and *rank* patient-relevant elements of dementia-care, and identify most/least preferred choices, are limited. The Analytic-Hierarchy-Process (AHP) may be one approach to elicit quantitative, choice-based preferences with PlwD, due to simple pairwise comparisons of individual criteria from a complex decision-problem, e.g. health care decisions. Furthermore, data on congruence of patient preferences with physicians’ judgements for PCC are missing. If patient preferences and physicians’ judgements differ, provision of PCC becomes unlikely. An understanding of patient preferences compared to physician’s judgements will support the implementation of truly PCC, i.e. state of the art dementia-care aligned with patient preferences.

**Methods:**

This mixed-methods-study will be based on the results from a previous systematic review and conducted in three phases: (I) literature-based key intervention-categories of PCC will be investigated during qualitative interviews with Dementia-Care-Managers (DCMs) and PlwD to identify actually patient-relevant (sub) criteria of PCC; (II) based on findings from phase I, an AHP-survey will be designed and pre-tested for face- and content-validity, and consistency during face-to-face “thinking-aloud”-interviews with PlwD and two expert panels (DCMs and physicians); (III) the developed survey will elicit patient preferences and physicians’ judgements for PCC. To assess individual importance weights for (sub) criteria in both groups, the Principal-Eigenvector-Method will be applied. Weights will be aggregated per group by Aggregation-of-Individual-Priorities-mode. Descriptive and interferential statistical analyses will be conducted to assess congruence of importance-weights between groups. Subgroup-analyses shall investigate participant-heterogeneities, sensitivity of AHP-results shall be tested by inclusion/exclusion of inconsistent respondents.

**Discussion:**

Little research is published on quantitative, choice-based preferences in dementia care. We expect that (1) PlwD have preferences and can express these, (2) that the AHP is a suitable technique to elicit quantitative, choice-based preferences among PlwD, and (3) to identify a divergence between patient preferences and physicians’ judgements for PCC. With the help of the AHP-technique, which supports systematic decision-making including multiple criteria, it may be possible to involve PlwD in future care decisions (patient participation) and ensure implementation of truly Person-Centered-Dementia-Care.

**Trial registration:**

Approval of the study was granted by the Ethics Committee at the University Medicine Greifswald the 09Apr2021 (Reg.-Nr.: BB 018–21, BB 018-21a, BB 018-21b).

## Background

With aging populations, age-associated diseases, such as dementia, represent a challenge for public health and health care systems worldwide [1]. According to findings from the *Global Burden of Disease Study 2019*, Alzheimer’s disease (AD) and other dementias were the fourth leading cause of death globally in the age groups 75 years and older, causing 5.6 (2.6–12.2) percentage of Disability Adjusted Life Years (DALYs) [2] and an estimated 1.55 (0.35 to 4.54) million deaths globally in 2019 [3]. The accelerated approval of aducanumab for people living with early stage AD by the *U.S. Food and Drug Administration* in June 2021 raised expectations for better pharmacological treatment of AD [4]. However, with refusal of marketing authorization by the *European Medicines Agency* in December 2021 [5], confidence in a soon widely available pharmaceutical treatment of AD has declined. Currently, no curative treatment for all People living with Dementia and Mild Cognitive Impairment [hereinafter commonly referred to as ‘PlwD’] exists. PlwD need a timely differential diagnosis [1, 6] and care, which ensures a high Quality of Life (QoL) [7].

According to the Alzheimer’s Association Dementia-Care-Practice-Recommendation, a person-centered focus is the core of quality care in dementia [7]. *Person-Centered Care* (PCC) has over the years been included in many countries’ national guidelines and dementia plans [8–14]. It challenges the traditional clinician-centered or disease-focused medical model to instead suggest a person-customized model of care [15–18]. The strategy of the PCC-model includes to incorporate personal knowledge and individual experiences of the PlwD, to conduct meaningful activities, to make well-being a priority, and to improve the quality of relationships between the health care professional and the PlwD [19–23]. Person-customization in PCC requires information about patient preferences [17, 18]. In dementia, some evidence about patient preferences exists. However, evidence about preferences elicited through quantitative, in particular choice-based preference methods is limited [24, 25]. A recent literature review focused on *decision-making tools* with PlwD from different countries by Ho et al. [26], found that earlier studies applied often qualitative methods and Likert-type scales. Harrison Dening et al. [27] elicited preferences from dyads during qualitative interviews. Van Haitsma et al. developed an extensive Likert-scale based *Preferences for Everyday Living Inventory (PELI)* for elicitation of preferences in community-dwelling aged adults [18]. These methods, however, fall short to *quantify, weigh* and *rank* patient-relevant elements of care, to measure their relative importance and identify most/least preferred choices. Such information can be assessed with quantitative, choice-based preference measurement techniques from Multi-Criteria Decision Analysis (MCDA) [28]. Groenewoud et al. [29], applied a quantitative, choice-based preference measurement tool (Discrete Choice Experiment (DCE)) focused on relevant aspects of outpatient care and support services for people with AD from the Netherlands, however with patient representatives and not patients themselves. Other MCDA-techniques commonly used in health care include Best-Worse-Scaling (BWS) [30] and the Analytic Hierarchy Process (AHP) [31, 32]. DCEs, depending on the number of choice sets included (full vs. fractional factorial design), usually include less, however cognitively more challenging questions. Depending on the number of elements included, the AHP may require to ask many questions. BWS distinguishes between three basic cases; object scaling (case 1), attribute or profile scaling (case 2) and multi-profiling (case 3), each case including various experimental designs, number of choice sets and questions. Hence, in BWS, the cognitive demands of included questions increases with each case [30]. For elicitation of patient preferences among people with cognitive impairments, the AHP has been suggested, as it may be more feasible than other MCDA-techniques, due to the simple pairwise comparisons with only two individual aspects of a complex decision problem [33].

Whether the challenges of the AHP can be handled by people living with Mild Cognitive Impairment or early to moderate-stage dementia is, to the best of our knowledge, still to be investigated [31]. Additionally, patient preference data elicited through quantitative, choice-based preference measurement tools in dementia from Germany are, to the best of our knowledge, missing entirely. Knowledge about patient preferences can inform provision of care that is most preferred by PlwD and avoidance of less preferred care, which is expected to have a positive effect on the life and care situation of PlwD, as well as to reduce the pressure on the health care system with limited resources by a prioritization of most preferred care options [28]. Additionally, the alignment of patient preferences with physicians’ judgements for person-centered dementia care has, to the best of our knowledge, not been investigated. Earlier studies of patient preferences versus physicians’ judgements in other indication areas found that experts’ judgements do not correlate well with subjective preferences of patients [34]. Knowledge about physicians’ judgments and their alignment with PlwD’s preferences is important, as physicians make decisions for their patients, are responsible for the diagnosis and monitoring of cognitive decline in their patients, and the provision of person-centered dementia care, i.e. state of the art dementia-care aligned with patient preferences.

## Methods/design

### Overall objectives of the PreDemCare-study

The aim of this study is to develop and apply a quantitative, choice-based preference measurement tool for Person-Centered Dementia Care. This entails

1. To identify patient-relevant (sub) criteria of Person-Centered Dementia Care for Development of an AHP-decision hierarchy with both patients and clinical experts.
2. To design and pre-test a dementia-friendly AHP-survey by an assessment of face- and content validity, as well as internal consistency with both patients an clinical experts.
3. To elicit patient preferences and physicians’ judgements for Person-Centered Dementia Care.
4. To analyze the congruence of PlwD and physician preferences for person-centered dementia care.
5. To identify preference and judgement patterns for person-centered dementia care associated with certain patient and physician characteristics.

### Setting & participants

Community-dwelling adults ≥60 years of age with an indication of Mild Cognitive Impairment (MCI) and early to mid-stage dementia in Mecklenburg-Western Pomerania, Germany, will be invited as participants in this study. Additionally, clinical experts including dementia-specialized nurses, so-called *Dementia Care Managers (DCMs)* [35, 36], and physicians from different specialties relevant in dementia-care, will be invited.

### Study design & methods

**Fig. 1:**
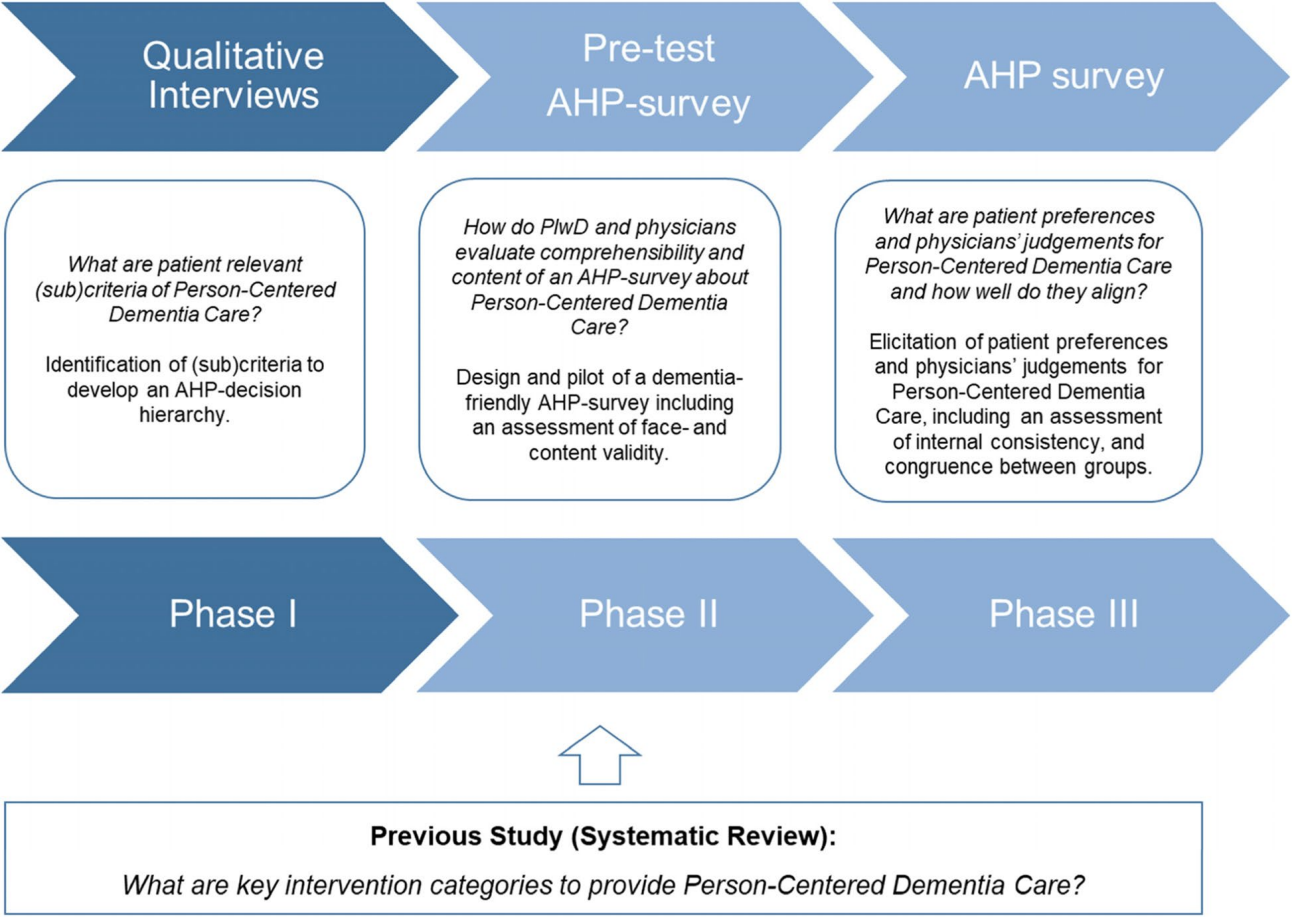
The mixed-methods design for the *PreDemCare*-study. Note: Own illustration developed oriented in Gilbert AW, Jones J, Stokes M, Mentzakis E, May CR. Protocol for the CONNECT project: a mixed methods study investigating patient preferences for communication technology use in orthopaedic rehabilitation consultations. *BMJ Open*. 2019 Dec 11;9(12):e035210. doi: 10.1136/bmjopen-2019-035210. The systematic review refers to Mohr et al. [41]. Abbreviations: AHP = Analytic Hierarchy Process, PlwD = People living with Dementia and Mild Cognitive Impairment

The *PreDemCare*-study [37] adopts a sequential mixed-methods-design [38] for final instrument development in line with core components in the design of a quantitative, choice-based preference study [39, 40]. An overview of the three phases of the *PreDemCare*-study, built upon a previous systematic review study [41], is shown in Fig. 1.

### Phase 0 – the basis: previous systematic review

In line with methodological recommendations for the design of a quantitative, choice-based preference study [32, 39, 40], we base the development of our study on a previous systematic review, which aimed to identify key intervention categories to provide person-centered dementia care from the published literature. As PCC at first is a theoretical concept [16, 42], we were interested in key intervention categories to *provide* person-centered dementia care. We identified nine key intervention categories: *social contact, physical activities, cognitive training, sensory enhancement, daily living assistance, life history oriented emotional support, training and support for professional caregivers, environmental adjustments,* and *care organization*. A detailed report of this previous study can be reviewed in Mohr et al. [41].

### Phase I: pre-study including qualitative interviews

For this formative qualitative pre-study phase, we orient our plan in recent guidelines by Hollin et al. [43].

#### Aim

The aim of phase I is to identify patient relevant (sub) criteria of PCC for PlwD to inform the development of an AHP-decision hierarchy. In consideration of the specific setting and context of community-dwelling PlwD in rural German Mecklenburg-Western Pomerania, a list of conceptual (sub) criteria will be developed prior to the interviews based on the key intervention categories of PCC identified by the previous systematic review [41]. This list will inform the qualitative data collection in phase I.

#### Sampling strategy & setting

Following recommendations for development of quantitative, choice-based preference measurement tools [39, 40, 43], we aim to include a diversity of perspectives and hence to conduct both 1) expert interviews with DCMs, and 2) patient interviews with community-dwelling PlwD. The interviews with the DCMs will be conducted at site. The interviews with PlwD are planned to be conducted in their homes or in day clinics. During the patient-interviews, the from literature identified conceptual (sub) criteria of Person-Centered Dementia Care will be presented to the participants with the aim to determine patient relevance. Thus, the literature-based (sub) criteria of Person-Centered Dementia Care will be reduced in number to keep the final AHP-decision hierarchy and -survey comprehensible [44], defined in further detail and will reflect the patient perspective. Study nurses as team members in active clinical trials at site (ClinicalTrials.gov identifiers: NCT04741932, NCT01401582, German Clinical Trials Register Reference No.: DRKS00025074) will function as gatekeepers to access PlwD for patient interviews. The inclusion of study nurses in the study design is deliberate, as they may be perceived as trustworthy by potential participants and previous research has highlighted the importance to include nurses during recruitment of study participants in dementia research [45]. The study nurses will emphasize the independence of this study from the clinical trials. None of the PlwD to be interviewed will know the main interviewers (WM, AR) personally on beforehand, but be aware of their professional roles. Participants who are eligible to enroll will be given a participant information sheet. All participants will be asked to provide written informed consent, which can also be provided by a legal guardian. On receipt of consent, the participant will be recruited into the study. All participants will be offered a copy of the consent form and a copy will also be saved in the project file. The same process will be applied for study phases II and III.

#### Sample size

For the expert interviews in phase I, *n* = 2 DCMs with a specialization and many years of work experience in dementia care will be interviewed. In this formative study phase we will aim to interview *n* = 10 PlwD, selected by purposive sampling [46] by aforementioned strategy. The sample size for the patient interviews has been determined based on the expected saturation point [46] and expected severely restricted access to patients due to the SARS-CoV2-pandemic. The latter i.a. includes ethical reflections in the study team to limit the risk associated with contact for both the vulnerable patient group and team members. All interviews will be conducted under adherence to a strict hygiene strategy developed at site.

#### Eligibility criteria

Eligibility criteria for PlwD for all phases are depicted in Table 1. To ensure a comfortable and non-stressful interview situation, PlwD can invite their informal CGs as silent supporters during interviews. It will however be communicated that the informal CGs shall not act as proxies and should not answer questions on behalf of the PlwD, if possible.

**Table 1:** Inclusion / exclusion criteria People living with Dementia in the *PreDemCare*-Study

Inclusion criteria	Exclusion criteria
Patient with indication of mild cognitive impairment or early to mid-stage dementia	Patient with no indication of mild cognitive impairment or early to mid-stage dementia
Age: ≥ 60	Age: < 60
Language skills: capable to understand written and oral German	Language skills: not capable to understand written and oral German
Written informed consent provided. This can also be provided by a legal guardian.	No written informed consent provided by either patient or legal guardian.

#### Data collection

We aim to conduct all interviews with two interviewers. Subject to participant consent, all interviews with PlwD will be audio recorded under consideration of participants’ privacy, i.e. the recording will be started after introduction of the participants. Field notes will be taken. The average interview time is expected to be approximately 60 minutes. We will use a self-developed semi-structured interview guide, oriented in [47], to ensure an efficient structure of the interview, whilst at the same time give the participants room to express themselves freely. The literature derived conceptual (sub) criteria and their descriptions will be translated to German and printed on cards in A5 format. In addition, the cards will include graphics to visualize the sub-criteria. In line with methodological recommendations for the design of a quantitative, choice-based preference study [32, 39, 40], the cards will, prior to the patient interviews, be reviewed by clinical experts in dementia care, the DCMs, for an early stage consideration of appropriateness and comprehensibility of the conceptual (sub)criteria. Suggestions by the DCMs will be noted in field notes and implemented directly. Oriented in [47], the cards will subsequently be presented to the PlwD as part of a ranking game during the interview to identify actually patient relevant (sub) criteria of Person-Centered Dementia Care. Furthermore, blank cards will be kept ready to add further (sub) criteria that may arise as important to the patients. Card game results will be documented in field notes and with pictures.

#### Data analysis

Card game results will be transferred to and analysed in Microsoft®Excel2019. The audio recordings from patient interviews will be transcribed verbatim. Names mentioned during the interview will be redacted from transcripts, e.g. with “XXX”, to ensure privacy. At least two reviewers will code transcripts with Qualitative Content Analysis [48, 49] in Microsoft®Word2019. The first interview will be coded independently by each reviewer based on the interview guide and the conceptual criteria identified from the literature, but allow for new categories to emerge. Subsequently, the reviewers will meet and discuss their codes and agree on a common strategy including a codebook and numerical code-identifiers for the remaining interviews. The codebook will be revisited after independent coding of the second interview to confirm the strategy by both reviewers. Subsequently, each reviewer will code the remaining interviews independently. Codes and emerging categories from transcript analyses, complemented with analyses of field notes and the card game results, will be discussed and agreed upon in a final common meeting between both reviewers and the research team. Specific coding software is not available. This study phase will entail a manifoldness of data sources (interview transcripts, field notes, card game results), which each have to be analyzed in different ways. To the best of our knowledge, no software is available that can incorporate all data sources respective to the planned analyses. The quality of data analyses in this study phase is deemed to be ensured by manual analyses in available software at site (Microsoft®Word2019 & Microsoft®Excel2019). Hence, the use of a specific coding software is not expected to yield an added benefit in comparison to cost for acquirement.

In accordance with recently published guidelines for formative qualitative research to support the development of quantitative preference studies [43], the findings of this qualitative phase are planned to be published.

### Phase II: pre-test AHP-survey in qualitative interviews

After the identification of patient-relevant (sub) criteria of Person-Centered Dementia Care based on the previous systematic review study and qualitative interviews with both experts and patients in phase I, an AHP-decision hierarchy and hereon-based first draft of the AHP-survey will be developed.

#### Aim

The aim of phase II is to pre-test the AHP-survey. Similarly to phase I, we orient ourselves in Hollin et al. [43] for conduct of this study phase.

#### The Analytic Hierarchy Process – a brief introduction

The AHP, a MCDA-technique, was developed in the 1970s by the mathematician Thomas Saaty [50, 51]. Its application in the health care sector was introduced in 1989 by Dolan et al. [52]. As a method of decision aid the AHP has mainly been used in the U.S. and Asia, a respective establishment in the Germany is discussed [32, 53, 54]. With the AHP, complex and unstructured decision problems, e.g. decisions related to health care, can be structured hierarchically. This shall aid the decision maker, e.g. patients, to achieve a plausible decision, by simple pairwise comparisons with a 9-1-9-point scale of elements in an overall decision problem. Based on the values assigned in the pairwise comparisons, a comparison matrix is developed [32]. By means of the Principal-Eigenvector-Method, individual importance weights are calculated [50, 51, 55]. Depending on how the AHP is applied, i.e. with individual representatives of a population or in a group decision setting, either the Aggregation of Individual Priorities-mode or Aggregation-of-Individual-Judgements-mode respectively are applicable for aggregation of individual importance weights. A more detailed description of the AHP and its application in the health care sector are outside the range of this study protocol for the *PreDemCare*-study, but can be found in Saaty [50, 51, 56–59], Dolan [52], Schmidt et al. [31], Danner & Gerber-Grote [60], and Mühlbacher & Kaczynski [32].

#### Expert panel 1

Initially, we aim to conduct an expert panel with *n* = 4 DCMs to review the first draft of the AHP-survey, including an assessment of appropriateness and comprehensibility, in preparation for subsequent patient interviews. Feedback from the experts will be documented as field notes in the survey and changes will be implemented immediately.

#### Patient interviews

For the patient interviews, we aim to recruit *n* = 10 patients, following the same recruitment process as outlined in phase I, for evaluation of the AHP-survey during in-depth interviews with the so-called “thinking-aloud”-technique [61]. Eligibility criteria are the same as depicted in Table 1, prior written consent is similarly to the in phase I outlined process required. As difficulties in recruitment of study participants are a known problem in dementia research [45], we aim to ask participants from phase I after completion of the interviews, whether they can be contacted again for the pre-test phase of the *PreDemCare*-study. Should this not meet the required sample size, recruitment of further participants will follow the strategy outlined in phase I. The pre-test is intended to assess the face- and content validity, including appropriateness and comprehensibility, as well as internal consistency of the AHP-survey [32, 62]. During the patient interviews, the PlwD, who can be accompanied by their informal CGs as silent supporters/ not as proxy raters, are asked to fill out the survey whilst “thinking aloud” with the researcher present. The participants will be asked to assess the formulations of the questions for their appropriateness and comprehensibility, as well as to provide information about their motivation when they answer the questions. Additionally, the PlwD will be asked about the layout of the survey and the appropriateness of the previously defined (sub)criteria. The interviews are expected to last approx. 60 min. It is aimed to complete all sections of the AHP-survey during pre-tests. Should participants express the wish to end the pre-test earlier, non-covered sections will be covered with other participants. Subject to prior written consent, interviews will be audio recorded in order to ensure completeness of the feedback provided by the patients on the AHP-survey. Considerations about privacy follow the same as outlined in phase I. Based on the feedback from the patients, the survey will be revised and adapted in terms of language, wording, structure and content on a continuous basis. Hence, patients’ feedback will be included in subsequent interviews until the patients have no further comments and no struggles with completion of the survey.

#### Expert panel 2

After the patient interviews, the resulting version of the survey will be assessed by a second expert panel with physicians to ensure incorporation of all relevant aspects, i.e. content validity of the survey. For the expert panel we aim to recruit a focus group of *n* = 5 physicians from different specialties relevant to the treatment and care of dementia diseases.

Based on the results from the patient interviews and the expert panels, two AHP-survey versions will be developed. One version will be for the patients and one for the physicians. The versions will be similar in content, but the physician survey will ask for the respondent’s professional judgement, i.e. their preferences as experts for Person-Centered Dementia Care.

### Phase III: Analytic Hierarchy Process, (assisted) paper & pencil survey

#### Aim

The aim of phase III is to elicit patient preferences and physician’s judgements for Person-Centered Dementia Care by application of the in phases I-II developed AHP-survey instrument.

#### Sampling strategy & setting

With regard to an appropriate sample size for an AHP-survey, no standard exists. IJzerman et al. [62] have applied the equation for sample size determination in conjoint analysis [63, 64] as a basis for the AHP. Following this, the sample sizes for this study will be determined based on a rule of thumb for Conjoint Analysis^1^ [64]. Hence, we aim to recruit *n* = 50 PlwD for study phase III. To investigate the correlation between patient preferences and physician’s judgements we plan to recruit *n* = 24 physicians, with the latter being the minimum number of participants needed based on the before outlined sample size calculation. Process for recruitment of PlwD will follow the same as outlined in study phase I, i.e. participants will mainly be recruited from clinical trials conducted at site (for a detailed description refer to ClinicalTrials.gov identifiers: NCT04741932, NCT01401582, NCT03359408, German Clinical Trials Register Reference No.: DRKS00025074) via study nurses. Experienced study nurses will be instructed to avoid selection bias during participant recruitment and hence obtain a sample, which is representative of the population intended to be analyzed, i.e. community-dwelling PlwD in the German Federal State Mecklenburg-Western Pomerania. If this should not yield enough participants, we aim to additionally recruit from the memory clinic at site [65]. Should these ways not yield enough participants, we plan to additionally recruit from external settings, e.g. day clinics and memory clinics. As participants will be community-dwelling PlwD from different areas in the federal state, this may enhance representativeness of the population and decrease selection bias. Eligibility criteria follow the same list depicted in Table 1, prior written consent is similarly to the in phase I outlined process required.

#### Data collection & analysis

The developed survey instrument will be provided as assisted paper & pencil survey for PlwD and non-assisted paper & pencil survey for physicians. The survey will consist of five sections:

1. A description of the study and an introduction to the criteria,
2. the first part of AHP-survey, i.e. pairwise comparison questions about criteria,
3. an introduction to the sub-criteria,
4. the second part of the AHP-survey, i.e. pairwise comparison questions about sub-criteria
5. a short sociodemographic survey including an evaluative question about the difficulty of the survey.

Initially, the participants will be presented with an explanation about the content of the survey and a description about the survey technique of the AHP. This includes a clarification about the hypothetical presentation of PCC, described by up to six criteria with each up to two sub-criteria. Apart from this, the participants will be presented with a laymen comprehensible description of each included (sub) criterion included in the survey. The pairwise comparisons will be presented in an appropriate context and explained. It is important that the participants receive enough information and details to be able to choose between the individual (sub)criteria. This will be supported by the inclusion of icons as visual aids for the different sub-criteria. Compared to other methods of preference elicitation, the AHP is expected to be particularly suitable for cognitively impaired patients, since the determination of patient preferences always takes only two individual aspects of a decision into account resulting in pairwise comparisons, which then are weighed against each other on the predefined AHP rating scale [31]. Aside from the PlwD, physicians will be asked to complete the survey based on their professional judgement, i.e. their preferences as experts for Person-Centred Dementia Care. In subsequent descriptive as well as interferential statistical analyses, the congruence between patient preferences and physician’s judgements will be investigated.

Sociodemographic and clinical variables will be analyzed with descriptive statistics. For the analysis of data collected with the AHP-survey, patient preferences will be elicited with coefficients of the included elements and a combination of both by application of the *Eigenvector*-method [50, 58]. This includes 1) local and global weights, i.e. relative preference weights, 2) estimates of the relative importance of the criteria, 3) data on consistency in response (consistency ratio for each pairwise comparison and respondent), 4) a sensitivity analysis by inclusion/ exclusion of inconsistent respondents, cf. Danner et al. [33] to assess, how changes in weights of criteria with a consistent model structure might influence the ranking of the different (sub) criteria, 5) a descriptive comparison of the patient preference/ physician judgement rankings of (sub) criteria for Person-Centered Dementia Care to assess the congruence and/or divergence, and finally depending on data completeness 6) univariable (e.g. independent paired t-tests, Mann Whitney-tests, one-way Analysis of Variance, Kruskal Wallis test, and Kendall’s correlation) as well as multivariable (e.g. multivariable regression models) statistical methods to assess eventual differences in preferences in correlation with respondent status (patient/ physician), socio-economic characteristics, and clinical status.

Analyses are planned to be conducted by usage of available software such as Expert Choice®Comparion [66], R (package: ahpsurvey [67]), and Microsoft® Excel 2019.

## Discussion

Little research is published on quantitative, choice-based preferences in dementia care [24, 25]. It is expected that PlwD have clear preferences for PCC and can express and name them. The AHP is expected to be a suitable technique for determination of care preferences among PlwD. The combination of qualitative sociological and quantitative mathematical research methods in participatory research is novel: there are only a few studies that determine the patients’ perspective in dementia care based on quantitative, choice-based preference elicitation tools [24, 25]. To the best of our knowledge, the application of such tools with cognitively impaired patients in Germany is one of the first of its kind. Similarly, a comparison of patient preferences versus physicians’ judgements in dementia in Germany is, to the best of our knowledge, one of the first of its kind.

To focus on a concept such as PCC for the experimental design of the AHP-decision hierarchy and -survey, may raise the question, whether a quantification of individual preferences can capture the core of PCC; an individualization of care and consideration of dementia as an individual process [19]. However and in contrast to other quantitative methods, which only allow to analyze aggregated data, the AHP allows for an evaluation of preferences on an individual basis for each participant, by which the individual process of dementia diseases may be considered [32]. Simultaneously, individual preferences can be aggregated and thus quantified for a group of decision makers, i.e. patients. Van Til & IJzerman [68] discussed the importance of patient preference consideration by regulatory and health policy decision bodies already in 2013. The authors highlighted the advantage of quantitative preference elicitation methods to measure patient preferences on a larger and representative scale, which in turn would allow decision committees to reflect the patient perspective in their regulatory/ health policy decisions [68]. The opportunity to elicit preferences in a large and representative sample of a patient population can improve the reliability and validity of preferences itself, and is necessary for comparability of preferences [68]. Knowledge about most/ least preferred health care options may further help to increase acceptance among patients and reduce the financial pressure on health care systems, as health policy makers could prioritize provision of those measures accepted and preferred by patients, i.e. patient−/ person-centered health care, and avoid less preferred options [28]. Still, some authors have questioned the stability and hence trust in predictability of patient preferences [69]. In this context, van Haitsma et al. [70] noted that preferences are based on the processing of needs, values, and goals, and hence can shift as the social environment or contextual circumstances change. To acknowledge that preferences may change could, in turn, contribute to consider the individual experience of dementia, an individualization of care, and thus fulfill the focus of PCC. However, PCC incorporates the necessity of relationship facilitation between the health care provider and patients [16]. It is questionable, how quantitative preferences can incorporate and represent a highly individual and complex process such as relationship facilitation. Nevertheless, PCC requires knowledge about patient preferences [16, 42]. Patients, including PlwD, are ‘experts by experience’ – hence, an incorporation of their perspective in care decision making is of importance [34]. Here, quantitative, choice-based preference elicitation tools, could form a powerful tool to consider the patient perspective on a larger and representative scale [68].

Analogous to the results of other patient preference studies [34], divergences between the preferences of PlwD and physicians’ judgements are expected. One may question the pros and cons to compare patient preferences and physicians’ judgements for Person-Centered Dementia Care, which is characterized by elements of nursing care and psychosocial support [41]. Hence, it may be questionable whether the comparison of patient preferences vs. nursing practitioners could be more appropriate, or in other words, whether physicians will be able to provide judgements about the importance of elements for dementia-care. A core element of PCC *is Shared-Decision Making* between the health care provider, including physicians, and the patient [41, 71, 72]. Here, the specific context of the German health care system needs to be considered; in the ambulatory health care setting, which community-dwelling people with mild cognitive impairment and early-moderate stage dementia i.a. navigate in, physicians, including both general practitioners and specialists, are essential in health care service provision, including the prescription of care services for these patients [73, 74]. A redistribution of tasks between specialized nurses and physicians, i.e. models of advanced nursing practice, are currently topic of research. But so far, such care models are not available in regular health care in Germany [74]. Due to the important role of physicians in German ambulatory health care service provision and the core element of *Shared Decision-Making* in PCC, an assessment of congruence between patient preferences vs. physicians’ judgements for Person-Centered Dementia Care, as planned for the *PreDemCare*-study, is considered appropriate. It is expected that physicians will be able to provide judgements about the importance of elements in dementia care.

The aforementioned pros/ cons of quantifying individual preferences and a comparison of patient preferences vs. physicians’ judgements in Person-Centered Dementia Care, may indicate how the expected results of our study can be used to improve standard dementia care procedures. The results may complement existing knowledge in national dementia guidelines, based on the results from clinical trials, with the patient perspective and hence support the implementation of truly person-centered, i.e. individualized care in dementia [16, 34, 42, 72]. If the AHP results to be a suitable technique to elicit patient preferences, this knowledge may be useful to enhance patient-physician communication, by greater focus on presentation of complex decision problems as simpler pairwise comparisons. Enhanced communication may facilitate relationships between physicians and patients, which, as mentioned before, is essential in the implementation of PCC [16, 42].

It has been purposefully decided by the research team to involve PlwD as ‘experts by experience’, as well as clinical professionals in the critical development-stages of the AHP-survey, to enhance both face and content validity [75]. The plan to access PlwD via study nurses as gatekeepers, and the invitation of informal CGs to join the interviews as silent supporters, as well as a rigorous informed consent process, which was reviewed and approved by the Local Ethics Committee at the University Medicine Greifswald the 9th of April 2021 (Reg.-Nr.: BB 018–21, BB 018-21a, BB 018-21b), shall ensure early consideration of consent and capacity [76]. Related to the planned sample sizes in the pre-study/instrument development phases (phases I and II), one may question the sufficiency of planned number of participants, which compared to usual sample sizes in other qualitative research may appear low. For conduct of the pre-study phases (including sample size estimation), we oriented ourselves in a recent publication by Hollin et al. [43], which entails guidelines for formative qualitative research to support the development of quantitative preference survey instruments. The authors emphasize that sampling in these study phases should not focus on number of units, but to collect actionable input for the development process, which needs a diversity of perspectives. They also underline that sampling adequacy in formative qualitative research may entail smaller samples than in general qualitative work, which given the limited study purpose may be adequate [43]. To complement suggestions by Hollin et al. [43], we orient ourselves in previous quantitative patient preference research, including works by first author AR, which reports similar sample sizes in the pre-study phase(s) [77–82]. With regard to include different perspectives, the inclusion of a third cohort of informal CGs as informants in the pre-study phases has been discussed in the research team. Previous research often included informal CGs as research participants as proxies/in dyads, including a recent DCE-based patient preference study by Chester et al. [83], despite findings of rater discrepancies between PlwD and their informal CGs [84–86]. In consideration of these previous findings and the goal of our study; to assess patient preferences with PlwD themselves, and not their informal CGs, which requires an AHP-experimental design that covers *patient*-relevant aspects, it has been decided in the research team to not include informal CGs as more than silent supporters of the PlwD in the pre-study phase. By inclusion of a planned total number of *n* = 31 participants in the instrument development phase including a variety of perspectives (*n* = 2 DCMs + *n* = 10 PlwD in the qualitative interviews for (sub) criteria identification, *n* = 4 DCMs for first expert panel, *n* = 10 PlwD for pre-tests of AHP-survey, *n* = 5 physicians for second expert panel), we align with recommendations [43] and previous research [77–82]. Furthermore, the planned sample sizes consider the mere practical obstacle to patient recruitment imposed by the ongoing SARS-CoV2-pandemic [87]. It is expected that the ongoing SARS-CoV2-pandemic will impact the conduct of the *PreDemCare*-Study. The research team has prepared for this by implementation of a strict hygiene strategy developed at site, which i.a. involves continuous testing of study personnel to interview/ survey the participants, and hopefully will make it possible to conduct this study in a reasonable time frame. To increase confidence in the final AHP-survey instrument, it is planned to publish reports about all study phases and hereby enhance transparency and confidence in the final AHP-survey instrument.

As this research will be conducted in one federal state of Germany, Mecklenburg-Western Pomerania, it may hence not be fully representative of the population and the health care services nationwide, which can be viewed as a limitation. However, participants will be recruited from several regions in the federal state and interviews/ surveys will be conducted in peoples’ homes, which enhances diversity of the study population in its respective region. Still, the exclusion of PlwD in nursing homes and people living with severe-stage dementia from the study population can be viewed as another limitation of this planned study. However, an early diagnosis and state of the art medical care aligned with patient’s preferences elicited through participatory research methods is necessary to ensure truly Person-Centered Dementia Care and a high QoL [6, 18, 20]. With the help of the AHP-method applied in the *PreDemCare*-Study, which supports systematic decision-making that takes multiple criteria into account, it may be possible to involve PlwD in future care decisions (patient participation) and ensure implementation of truly Person-Centred Dementia Care.

## Data Availability

Not applicable. For each phase of the study, a manuscript will be written and submitted to national and international conferences. Additionally, summaries in easy language will be developed and made available for patients and the public, .e.g. through dementia interest groups. All results from each phase of the study will be published in peer-reviewed journals. Links to research outputs will be made available on the *PreDemCare*-website, hosted by the German Center for Neurodegenerative Diseases e.V. (DZNE), available at: https://www.dzne.de/en/research/studies/projects/predemcare/.

## Abbreviations

AHP: Analytic Hierarchy Process
CGs: Caregivers
DCMs: Dementia Care Managers
MCDA: Multi-Criteria Decision Analysis
PlwD: People living with Dementia
PCC: Person-Centered Care
QoL: Quality of Life

## Footnotes

1 (NxTxA)/C ≥ 500, where *N* = number of respondents, T = number of choice sets per respondent, A = number of scenarios per choice set, and C = maximum number of levels
